# Alcohol-Induced Oxidative Stress and the Role of Antioxidants in Alcohol Use Disorder: A Systematic Review

**DOI:** 10.3390/antiox11071374

**Published:** 2022-07-15

**Authors:** Evangelia Eirini Tsermpini, Anja Plemenitaš Ilješ, Vita Dolžan

**Affiliations:** 1 Pharmacogenetics Laboratory, Institute of Biochemistry and Molecular Genetics, Faculty of Medicine, University of Ljubljana, 1000 Ljubljana, Slovenia; evangelia-erini.tsermpini@mf.uni-lj.si; 2 Department of Psychiatry, University Clinical Centre Maribor, 2000 Maribor, Slovenia; anja.plemenitas@ukc-mb.si

**Keywords:** alcohol use disorder, alcohol dependence, alcohol addiction, alcohol dehydrogenase, aldehyde dehydrogenase, oxidative stress, catalase, glutathione

## Abstract

Alcohol use disorder (AUD) is a highly prevalent, comorbid, and disabling disorder. The underlying mechanism of ethanol neurotoxicity and the involvement of oxidative stress is still not fully elucidated. However, ethanol metabolism has been associated with increased oxidative stress through alcohol dehydrogenase, the microsomal ethanol oxidation system, and catalase metabolic pathways. We searched the PubMed and genome-wide association studies (GWAS) catalog databases to review the literature systematically and summarized the findings focusing on AUD and alcohol abstinence in relation to oxidative stress. In addition, we reviewed the ClinicalTrials.gov resource of the US National Library of Medicine to identify all ongoing and completed clinical trials that include therapeutic interventions based on antioxidants. The retrieved clinical and preclinical studies show that oxidative stress impacts AUD through genetics, alcohol metabolism, inflammation, and neurodegeneration.

## 1. Introduction

Alcohol use disorder (AUD) is a highly prevalent, comorbid, and disabling disorder. Alcohol dependence (AD), abuse, dependence, or harmful use are under the spectrum of AUD [1]. AD is a common and debilitating psychiatric disorder, with high heritability, that causes a substantial global burden of morbidity and mortality [2,3]. AUD is associated with many physical and psychiatric comorbidities [4,5] and contributes substantially to global morbidity and mortality [6]. Five in one hundred deaths worldwide are due to the consumption of alcohol [7].

According to a status report in 2017 on global statistics regarding alcohol, 18.4% of adults had consumed more than 60 g of alcohol on one occasion in the past 30 days. Europeans consumed the highest amount of alcohol; however, the rate of alcohol consumption was lower in Mediterranean countries than in Northern and Eastern Europe. On the other hand, North Africans and Middle Eastern ethnicities had the lowest alcohol consumption per capita [2]. AUD is less common in women than in men, where the lifetime risk is more than 20% [1].

Although AUD is the most prevailing cause of dependence, with approximately 60 million cases worldwide [2], only 25% of patients seek help. The diagnosis is challenging, given that patients have an average daily life and usually only experience general symptoms such as depression, anxiety, and insomnia [1].

The Diagnostic and Statistical Manual of Mental Disorders (DSM-5) integrates the two DSM–IV disorders, alcohol abuse and AD, into a single disorder called AUD with mild, moderate, and severe subclassifications [8]. In addition to the DSM criteria, the alcohol use disorder identification test (AUDIT) screening test is also used in genetic studies to better characterize alcohol-related phenotypes [9]. AUDIT is a straightforward, practical, self-reported tool developed by the World Health Organization for the early detection of alcohol misuse [9,10].

### 1.1. Ethanol Metabolism

Ethanol metabolism occurs mainly in the liver in two steps, starting with oxidation to acetaldehyde and then oxidation of acetaldehyde to acetate [11].

**Figure 1:**
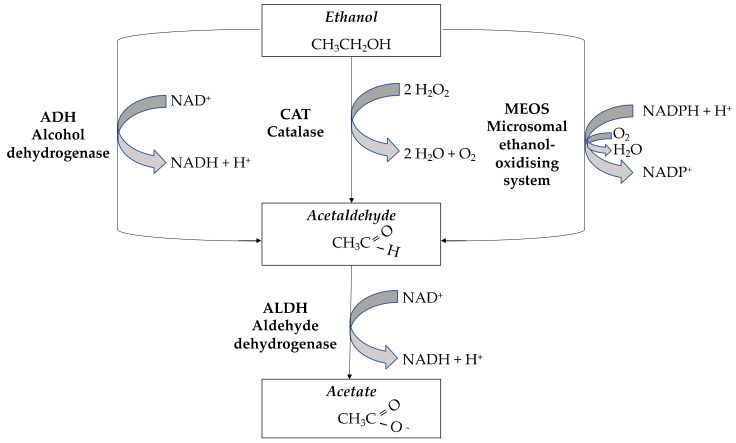
Ethanol metabolism.

As shown in Figure 1, three metabolic pathways participate in the first step of ethanol metabolism: alcohol dehydrogenase (ADH), catalase (CAT), and the microsomal ethanol oxidation system (MEOS). ADH and aldehyde dehydrogenases (ALDH) are phase I enzymes that catalyze the first and the second oxidation step, respectively [12,13]. ADH has a high affinity but low capacity for ethanol; therefore, CAT has a prominent role in heavy ethanol consumers, while MEOS is induced by chronic ethanol consumption [14]. The CAT pathway plays a central role in ethanol oxidation in the brain [15]. MEOS is involved in ethanol oxidation by the cytochrome P450 (CYP) enzymes including CYP2E1, CYP1A2, and CYP3A4 isoforms [16]. Increased ethanol metabolism rates may contribute to the development of AD: faster ethanol inactivation during long-term alcohol drinking may increase the motivation to consume more alcohol to maintain a desired level of ethanol at target sites [16].

Acetaldehyde is not the only reactive metabolite of ethanol metabolism. Reactive oxygen species (ROS) may also be produced, in particular as by-products of oxidation via CYP enzymes. ROS are then transformed into less reactive products by antioxidative enzymes, while phase II conjugating enzymes, such as glutathione S-transferases (GSTs), participate in detoxification of their reactive secondary products [17]. Ethanol and acetaldehyde levels depend on the activity of ADH and ALDH during detoxification, whereas GSTs regulate ROS levels via binding with glutathione. Thus, ROS production is related to alcohol metabolism to acetaldehyde and acetate and can potentially disturb cells’ oxidative balance and increase the risk of oxidative stress [18,19]. 

### 1.2. Alcohol and Oxidative Stress

Ethanol metabolism has been associated with increased oxidative stress [14]. Oxidative stress is defined as an imbalance between the production of free radicals and the antioxidant system [20]. The toxic effects of alcohol are mediated by oxidative stress through various mechanisms such as induction of oxidative damage, lipid peroxidation, crosslinks, DNA adducts, and DNA strand breaks [21]. At the cellular level, mitochondria are the primary source of ROS production. Alcohol-induced oxidative stress was shown to be related to the impairment of antioxidants including superoxide dismutase (SOD), CAT, and glutathione peroxidases (GPX) activities [22]. CYP2E1 generates reactive ROS during the catalytic cycle, which can cause oxidative stress, triggering lipid peroxidation, protein inactivation, increased cytokine production, mitochondria, and DNA damage leading to cell death [16]. Excessive oxidative stress damages all major macromolecule classes and affects several fundamentally critical cellular functions. Consequences that are especially detrimental to the proper functioning of the brain include mitochondrial dysfunction, altered neuronal signaling, and inhibition of neurogenesis [23]. In addition to its role in ethanol metabolism, CAT represents an important enzymatic system able to inactivate ROS and their by-products [16]. CAT enzyme activity is increased by chronic alcohol consumption, while a shorter consumption period decreases its activity. ALDH2 plays a crucial role in removing endogenous aldehydes, such as malondialdehyde (MDA) and 4-hydroxy-2-nonenal, produced by lipid peroxidation triggered by oxidative stress [24,25].

Ethanol is also metabolized to acetaldehyde by CYP2E1, thus producing ROS, including hydrogen peroxide (H_2_O_2_), which can also damage tissue [26,27]. It should be noted that more than half of acetaldehyde in the brain is attributed to CAT [28], as well as that that acetaldehyde is a very toxic product and can further trigger the production of ROS, which includes both reactive molecules and free radicals derived from molecular oxygen [11,27]. The abundance of acetaldehyde increases nicotinamide adenine dinucleotide phosphate (NAPDH) oxidase 2 (NOX2) expression and activity, which are crucial in the generation of acetaldehyde-induced mitochondrial ROS. Acetaldehyde prompts ROS overproduction and can lead to oxidative stress [29]. Moreover, oxidant levels can be increased through mitochondrial oxidative phosphorylation, cellular activation of NADPH oxidase/xanthine oxidase (NOX/XOX), nitric oxide synthase (NOS), and a copper/iron-catalyzed Fenton–Weiss–Haber reaction of H_2_O_2_ [26]. The exact mechanism of action of alcohol-induced oxidative stress is not fully known. However, oxidative damage to mitochondria and disturbances in cell functions are initiated by alcohol consumption. Haorah and colleagues hypothesized and indicated that the generation of ROS and NO in human neurons during ethanol metabolism by ADH and CYP2E1 is due to the activation of NOX/XOX and inducible NOS (iNOS) by acetaldehyde [26].

Oxidative stress is fundamental to the etiology of AUD [30]. ROS induces oxidative stress in cells and potentially damages neurons. Endogenous antioxidants, such as SOD, GPX, and reduced glutathione, are essential for ROS elimination. SOD inactivates radical superoxide and forms H_2_O_2_. In the GPX reaction, glutathione is an electron donor for H_2_O_2_ and subsequently removes ROS [31]. Glutathione levels in the brain decreased after acute and chronic alcohol consumption [32]. Chronic alcohol consumption also impacts GST, but studies’ findings are inconsistent [17].

Chronic excessive alcohol consumption results in inflammation in multiple organs including the brain. Prolonged oxidative stress in the brain is associated with neuroinflammation and neurodegeneration [33]. Neuroinflammation contributes to alcohol-related cognitive dysfunction and behavioral alterations, but understanding the mechanisms by which alcohol triggers inflammation in the brain is limited [34]. Alcoholism can be considered an inflammatory condition, as peripheral endotoxemia may lead to brain inflammation and increased secretion of pro-inflammatory cytokines such as tumor necrosis factor-alpha (TNF-α), interleukin (IL)-1β, IL-6, and interferon γ [35]. Alcohol rapidly diffuses through the blood–brain barrier, alters neurotransmission, and contributes to neurodegeneration and impaired regeneration by activating microglia and astrocytes [34]. The activation of astrocytes may be mediated by the Toll-like receptor-4 pathway (TLF-4), resulting in the recruitment of downstream signaling molecules and cytokine secretion [34,36].

### 1.3. Comorbid Mental Disorders and AUD

Clinical and epidemiological studies have shown that AD individuals have a significantly higher risk of other co-occurring mental disorders than the incidence of mental disorders in the general population [37,38]. Animal and human studies confirmed a link between oxidative stress and anxiety, depression, and AUD. Oxidative stress might also be involved in the etiology of neuropsychiatric diseases by causing accelerated telomere shortening, mitochondrial dysfunction, inflammation excitotoxicity, and influence on neuronal signaling [23]. Oxidative stress profoundly affects behavior, similar to what is observed with chronic alcohol intake, indicating that it may be the likely underlying mechanism of alcohol behavioral impairments [39]. The most common co-occurring psychosymptomatology in AUD are depressive and anxiety disorders, attention-deficit hyperactivity disorder, post-traumatic stress disorder (PTSD), aggression and personality disorders, and dependence on other psychoactive substances [37]. Studies have indicated that the co-occurrence of AUD and depressive disorders are associated with greater severity and a worse prognosis for both disorders [40]. Among individuals who have schizophrenia or schizoaffective disorder, AUD is common, contributing to worse outcomes [41]. Personality disorders characterized by impulsivity and affective dysregulation (specifically antisocial and borderline personality disorders) may be associated with severe consequences and co-occurrence with AUD [42]. Clinical and epidemiological studies show that having either an anxiety- or alcohol-related diagnosis elevates the prospective risk of developing the other disease, supporting that these conditions share underlying, mutually exacerbating neurobiological processes [43]. AUD renders chronically heavy drinkers vulnerable to direct alcohol toxicity and various comorbidities and puts aging drinkers at risk for developing cognitive decline and, possibly, dementia [44]. AUD is a potent risk factor for suicide, with a significant association persisting after accounting for confounding factors [45].

This review aimed to summarize the findings on AUD and oxidative stress. We searched the PubMed.gov, genome-wide association studies (GWAS) catalog, and ClinicalTrials.gov databases to systematically review the data on clinical and genetic factors related to AUD and oxidative stress.

## 2. Methods

We searched the available information in the PubMed.gov (https://pubmed.ncbi.nlm.nih.gov/, accessed on 27 February 2022), GWAS catalog (www.ebi.ac.uk/gwas/, accessed on 28 February 2022), and ClinicalTrials.gov (https://clinicaltrials.gov/, accessed on 23 February 2022) databases. We used different keywords while searching these three databases, because they have different search strategies. In PubMed, searches can be performed using keyword combinations. The GWAS catalog only allows searching one keyword at a time, while ClinicalTrials.gov only allow searches for “a condition or disease” and “other terms”.

For the PubMed.gov search, we used the following combination of keywords: alcohol use disorder, alcohol dependence, alcohol addiction, alcohol abstinence, oxidative stress, genomics, genetics, genes, polymorphisms, and oxidative stress genes.

All of the available published articles and clinical trials registered up to 28 February 2022, were included in our review. Book chapters, books, and dissertations as well as articles without available full texts were excluded. Only English papers on the adult human population older than 18 years were retrieved. Studies on animal models were also included. We manually screened 109 articles on PubMed and retrieved 65 papers exploring oxidative stress in AUD. Finally, 49 papers published in the last ten years were included in this review. We also retrieved relevant studies from the 13 review articles identified through our search.

In addition, we searched the GWAS catalog to identify GWAS studies performed on AUD and AD. Three epigenetic studies emerged using the keyword, alcohol use disorder, but none of them were related to oxidative stress. By using the keyword, alcohol dependence, 45 studies emerged. Three studies reported no statistically significant results, and six reported statistically significant results but did not include oxidative stress genes. Ten studies included AD in combination with schizophrenia (1), bipolar (1), pancreatitis/cirrhosis (2), psychiatric disorders in general (1), depression (2), sexual behavior (2), and heroin and other substances (1). Hence, only nine studies were included in the review. Additional reports relevant to the research topic also emerged from these articles.

We also included information on clinical trials. The ClinicalTrials.gov database search was performed using the keywords, alcohol use disorders and alcohol dependence, in combination with oxidative stress. We identified 26 studies, 10 of which were duplicates. Therefore, we manually screened 16 trials, which were included in this review. To minimize the publication bias of our systematic literature search, we reported the positive findings and the limitations of specific therapeutic approaches.

**Figure 2:**
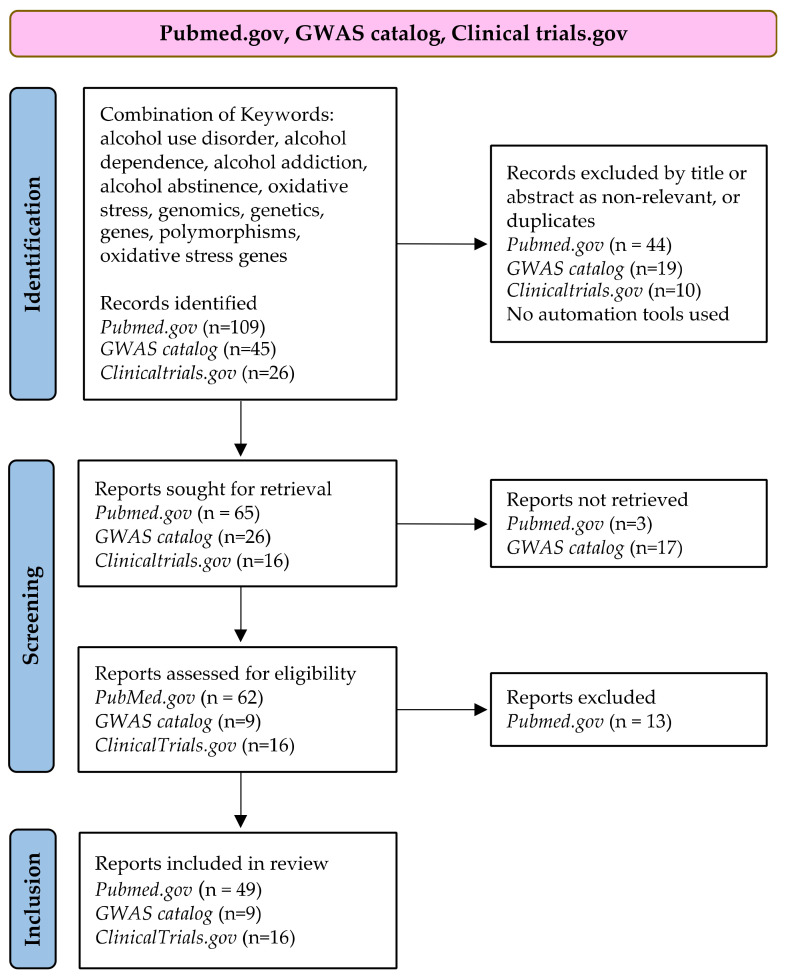
PRISMA flow diagram.

A PRISMA flow diagram of the systematic search for studies and clinical trials on AUD used for this review, following Page et al. [46], is shown in Figure 2.

## 3. Results and Discussion

### 3.1. AUD and Oxidative Stress in Preclinical and Clinical Studies

AUD is associated with oxidative stress and, subsequently, antioxidant mechanisms are affected. Endogenous antioxidants, such as SOD, GPX, and glutathione, are essential for ROS elimination. CAT represents an important enzymatic system able to inactivate ROS and their by-products [16]. Free radical products in the liver during alcohol oxidation have been shown to increase the serum levels of MDA [47].

An animal study using crossed high alcohol-preferring mice reported that chronic voluntary drinking caused anxiety-like behaviors. Alcohol increased the expression of neuroinflammation markers and induced oxidative stress and endoplasmic reticulum stress [48]. Clinical and rodent studies have studied the acute and chronic effects of alcohol on glutathione levels. Acute alcohol exposure in humans and rodents does not regulate glutathione or GPX levels. In contrast, chronic alcohol intake consistently reduces glutathione or GPX levels in the brain and plasma and alters GST in the brain, blood, and saliva [49,50].

A study comparing oxidative stress in AD patients during hospitalization showed lower SOD levels at discharge. In comparison, GPX and ferric reducing antioxidant power presented higher levels that indicate reversion of alcohol effects over oxidative stress parameters [51]. Another investigation of the total antioxidant activity of human serum blood carried out by voltametric, amperometric, and chemiluminescent methods revealed that the serum total antioxidant activity of patients with AD was lower than the total antioxidant activity of healthy donors [52]. A previously published study reported an oxidative stress-related increase in Mn-SOD in AD patients [53]. A study investigating the relationship between AD and oxidative status reported that serum MDA levels of AD patients significantly increased compared with controls and decreased after abstinence. Serum CAT did not return to normal levels two weeks after abstinence. They also reported that CAT activity was significantly correlated with MDA levels [54]. The correlation between alcohol withdrawal severity and two oxidative stress markers, MDA and SOD, revealed that alcoholic patients encountered high oxidative stress, since MDA levels were significantly elevated. SOD activity was significantly decreased in AD patients. Their clinical withdrawal severity during early withdrawal was correlated with the extent of lipid peroxidation [55]. During early detoxification, oxidative stress markers showed marked oxidative stress in AD patients without severe liver disease. The attenuation of a raised MDA level and a lowering of CAT activity appeared after 1 week of detoxification. Another finding was that AD patients did not scavenge free radicals as readily as controls [22]. A clinical study investigating oxidative damage to plasma proteins in patients with chronic AD and the effect of smoking as a confounding factor reported that systemic oxidative stress in chronic AD is attributed mainly to alcohol consumption. In contrast, smoking may act synergistically by overwhelming the plasma antioxidant defenses, exacerbating the oxidative stress process [56].

Another study investigated how oxidoreductive blood balance could affect demand for energy substances, such as alcoholic beverages, in AD individuals and the severity of their AD and risk of drinking relapse. They measured aldehyde products of lipid peroxidation (i.e., MDA and 4-hydroxy-2-nonenal), nitric oxide metabolites, total antioxidant status, and activities of GPX, SOD, and glutathione reductase in the blood. The authors reported that the risk of alcohol drinking relapse was lower in patients with an above-median initial blood concentration of nitric oxide metabolites and total antioxidant status. The oxidative stress parameters correlated with AD severity markers [57].

Based on animal and human studies, we can conclude that acute alcohol exposure has no critical influence on oxidative stress, proven through the glutathione or GPX level. On the other hand, chronic alcohol consumption consistently reduces glutathione, GPX, and GST levels in the brain. This can be related to neurochemical processes involved in neurotoxicity and pathogenesis in AUD. CAT, a crucial enzymatic system able to inactivate ROS and their by-products, did not return to normal levels after a short period of abstinence. In addition, withdrawal severity was correlated to higher oxidative stress and the extent of lipid peroxidation. Higher oxidative parameters were also correlated with the severity of AD and a higher risk of drinking relapse.

Table 1 presents an overview of clinical studies on AUD and oxidative stress, while Table 2 summarizes review articles on the topic.

### 3.2. Genetics of Alcohol-Related Disorders

ADH and ALDH enzymes exist in different isoforms with different characteristics and are encoded by different genes [12]. These genes have many alleles; some may differ in only one position, where a single nucleotide substitution takes place [74]. The genetic variability of *ADH* and *ALDH* impacts the conversion of acetaldehyde to acetate and affects the rate of alcohol metabolism in the liver, leading to different phenotypes [12,13,74]. For example, there is evidence that specific *ADH* and *ALDH* variants can cause rapid oxidation of ethanol, and slower oxidation of acetaldehyde, respectively. As the accumulation of acetaldehyde can lead to unpleasant symptoms, such as flushing syndrome and nausea, *ADH*’s and *ALDH*’s genetic variability have a protective role in AUD [12,13]. Coding variations that alter enzyme activity and noncoding variations that affect gene expression of *ADH* and *ALDH* have been associated with alcohol metabolism [12,74]. It should also be noted that the distribution of polymorphisms in genes involved in alcohol metabolism differs among different ethnicities and races [75].

Seven human *ADH* genes have been identified (i.e., *ADH1A*, *ADH1B*, *ADH1C*, *ADH4*, *ADH5*, *ADH6*, and *ADH7*) located in chromosome 4. *ADH1A*, *ADH1B*, and *ADH1C* are the most important in ethanol oxidation in the liver [12]. *ADH1C* exhibits high activity for ethanol oxidation to acetaldehyde, therefore, having a significant role in ethanol catabolism [3]. Regarding genetic variability, *ADH1B* has three alleles, ADH1B*1, ADH1B*2, and ADH1B*3, which encode the β1, β2, and β3 subunits, respectively. ADH1B*1 is considered the reference allele, whereas ADH1B*2 (also known as rs1229984) is more common in Asian and ADH1B*3 (also known as rs2066702) in African populations. In the presence of ADH1B*2 and ADH1B*3, the NAD+ coenzyme is released more quickly than in ADH1B*1 during alcohol oxidation [12,74].

Three alleles also exist for *ADH1C*, i.e., ADH1C*1 (or rs698), ADH1C*2, and ADH1C* Thr352. ADH1C*Thr352 and ADH1C*2 are in high linkage disequilibrium (LD) and are usually inherited together. It has been shown that the ADH1C*2 allele is associated with lower rates of ethanol oxidation, whereas higher rates of ethanol oxidation are related to the presence of the ADH1B*2 and ADH1B*3 alleles [12,74]. *ADH4* has been associated with increased levels of alcohol consumption. *ADH5* and *ADH7* are involved in first-pass metabolism, whereas little is known about the role of *ADH6* [74].

In contrast to *ADH*, *ALDH* genes can be found in different chromosomes. Although ALDH1A1, ALDH1B1, and ALDH2 are crucial in acetaldehyde oxidation, ALDH2 in mitochondria is the most significant in this process [74]. The *ALDH2* gene is highly polymorphic, and the ALDH2*2 allele, known as rs671, encodes an inactive form of the enzyme, which leads to the accumulation of acetaldehyde [58,74]. ALDH2*2 has a higher frequency in East Asian populations and is strongly associated with the risk of alcoholism [74]. This polymorphism alters the drinking behavior and risk of AUD in East Asian populations, as it causes unpleasant flushing responses after alcohol consumption and prevents excessive alcohol intake [24]. The inactive form of ALDH2 is associated with reduced alcohol consumption in healthy people [76,77]. Association studies have consistently shown that the inactive form of ALDH2 decreases AUD risk [78]. ALDH2 is a critical player in removing endogenous aldehydes, such as MDA and 4-hydroxy-2-nonenal, produced by lipid peroxidation triggered by oxidative stress [24,25]. The activity of ALDH2 to catalyze the toxic endogenous aldehydes protects individuals from various forms of organ damage related to oxidative stress.

Studies using BXD recombinant inbred (RI) mice investigated genes that are associated with alcohol sensitivity and a predisposition to alcoholism to understand the underlying mechanisms of neurotoxicity in response to ethanol [79]. In evaluations of the genetic susceptibility to ethanol consumption and withdrawal, strains showed contrasting behavioral responses to ethanol, since the BXD RI strains showed that C57BL/6J (B6) inbred mice were able to drink large amounts of ethanol voluntarily, while DBA/2J (D2) mice showed improved withdrawal to acute ethanol [80]. QTL analyses in DBA/2J (D2) and C57BL/6J (B6) mouse strains identified several chromosomal regions involved in risk for acute ethanol withdrawal and behavioral responses to ethanol [81,82].

Although the exact mechanism of AUD is still unclear [3], environmental factors, such as patterns of alcohol consumption, family history, and early life stressors, play a significant role in the development and the severity of AD [3,13,83]. Additionally, a meta-analysis of twin and adoption studies demonstrated that the heritability of AUD is approximately 50% [84], indicating the strong influence of genetic factors on the emergence and severity of the disorder. Linkage studies have identified genetic loci on chromosome 4, which contains *ADH* genes, but not clearly defined genetic risk variants associated with AUD [13]. Candidate gene and GWAS highlight the significant role of the *ADH* gene cluster on AD risk, given that the majority observed strong associations with *ADH* and *ALDH* [13]. Polymorphisms in *ADH* and *ALDH* genes are the most commonly identified, often having a protective role against AUD development [13,85]. However, candidate gene studies and GWAS have identified only limited genetic loci, concluding that additional unidentified loci might play an important role in AUD [86].

### 3.3. Candidate Gene Studies on AUD and Oxidative Stress

Candidate gene studies can identify small-effect genetic risk variants associated with AUD [13]. Searching the literature, only a limited number of studies focus on the genetic variability of oxidative stress genes such as *CAT* and *GST*s.

Although there is evidence that CAT activity and levels are related to alcohol consumption [87,88], the study findings regarding the potential impact of *CAT* genetic variability are conflicting. *CAT* rs1001179 showed no association with AUD [28] in a cohort of African Americans. In a Caucasian cohort, the frequency of rs1001179 T allele carriers was higher in AD patients and more elevated in AUDIT scores [89].

Regarding the role of the genetic variability of *GSTM1* and *GSTT1* in alcohol consumption, there is one study that included patients with hepatocellular carcinoma, indicating the influence of the *GSTM1* null genotype in the high ethanol intake [90].

*GSTP1* rs1695 has also been associated with alcohol consumption. More specifically, the frequency of the GG genotype was higher in AUD patients than in healthy individuals [21].

A very interesting study that investigated the influence of the genetic variability of four alcohol metabolizing genes (i.e., *ADH1B*, *ALDH2*, *GSTM1*, and *GSTT1*) in AUD indicated that *ADH1B* had a protective effect against alcohol consumption. In addition, ALDH*2/*2 was present only in patients, and the *GSTT1* null genotype was associated with AUD [11].

A study-wide significant association between *ADH1* rs1229984 with alcohol consumption and flushing was also observed in a European Australian population. Interestingly, when controlling for this SNP, more associations emerged. *ADH1B* rs1042026 was associated with alcohol intake, whereas *ADH1C* rs1693482 and *ADH5* rs1230165 were associated with alcohol consumption [91]. *ADH1B* rs1229984 had a protective effect against AD in Europeans and African Americans [92], while it was significantly associated with drinking behavior in the Japanese population [93]. A strong association of this polymorphism with the DSM-IV symptom count and the maximum number of drinks was also observed in European Americans [94]. Male Japanese AD patients had higher ADH1B*1 allele frequencies than the controls [67], and male Jewish Americans with ADH1B*2 showed lower rates of alcohol consumption and more unpleasant reactions [95]. Association was also observed with AD for *ADH1C* rs1614972 in a cohort of European AD patients and healthy controls [96]. In a replication study, which investigated the influence of 43 polymorphisms that emerged from previous GWAS, indicated that *ADH1C* rs1614972 was associated with AD [96].

Birley et al. covered a fragment of 497 kb of the ADH region of chromosome 4 in a large cohort of Northern and Southern Europeans. According to their findings, *ADH1A* rs931635, rs1229967, rs1618572, and rs1230025 had an increasing effect, and rs2276332 had a decreasing impact on early-stage metabolism. rs3857224 and rs3762894, located in the *ADH6*–*ADH4* intergenic region, were related to early stages of metabolism. *ADH1B* rs2018417, rs1229985, rs17033, and rs1789877 had an early-increasing effect, a late-increasing effect, an early-decreasing effect, and a late-increasing effect, respectively, on alcohol oxidation [97].

ADH2*1 was more frequent in Japanese [98] and Mission Indians AUD patients than in healthy controls [99]. At the same time, the ADH2*2 allele had a protective effect against AUD in young male New Zealand Maori, even when ALDH2*2 was absent [100]. ADH2*3 was associated with AD and a lower number of maximum drinks [99].

Edenberg et al. found significant associations between *ADH4* rs1984362, rs4699718, rs3762894, rs4148886, rs4699714, rs7694646, rs1126672, DWSHpy188I, rs1042364, rs1042365, rs2602866, and rs2602846 and alcoholism [101]. The association of *ADH4* rs1042364 was replicated in AUD patients from Central-West Brazil [102]. In addition, *ADH4* rs1800759 was related to the early stages of metabolism, whereas *ADH4* rs2032349 and rs1573495 were associated with the elimination rate [97].

*ALDH1A1* rs8187974 [94] and *ALDH1A1* rs348479 and rs610529 [103] were associated with AD in European Americans and Finnish, respectively. Association between the AUDIT scores and *ALDH1A1* rs348449, rs610529, and rs348479 polymorphisms rs610529-rs2288087 haplotype, have also been observed in Finnish males. In addition, the authors observed a strong association between rs348449 and problematic drinking [103]. Lifetime AD diagnosis and high levels of alcohol consumption were also reported for ALDH1A1*2 in a cohort of African or Indian ancestry [104].

ALDH*2/*2 was associated with AD in Central Indians [11], whereas ALDH2*1 was more frequent in Korean [105] and Japanese patients with AUD [98]. However, a recent study indicated that Japanese patients with AD had higher allele frequencies of ALDH2*2 than healthy individuals [58]. A strong association between *ALDH2* rs671 and drinking behavior has also been observed [93,106] in Taiwanese and Japanese patients. Finally, *ALDH2* rs2238151 had a protective effect against AD in the Han Chinese population [107].

It is crucial to mention that findings from meta-analyses indicate the involvement of *ALDH2* rs671 [108,109,110] and *ADH1C* rs698 [109] in Asian patients with AD, as well as the protective role of *ADH1B* rs1229984 against Asian AUD patients [109,110,111].

Lastly, it is essential to mention a clinical trial that investigated the influence of the genetic variability of alcohol metabolizing enzymes in 101 AUD patients treated with naltrexone. According to the findings, *ADH1C* rs698 and *ALDH2* rs671 were associated with better responses, whereas AUD patients with *ADH1B* rs2066702 had lower naltrexone response rates [112].

Candidate gene studies that identified genes and genetic variations associated with a high risk of developing AUD provided valuable information, as the products of these genes are involved in physiological and psychological behavioral processes such as the reward system. In addition, AUD development results from interactions between genetic and environmental factors as well as epigenetic modifications that alter gene expression. Currently, most candidate gene studies indicate that *ALDH2* rs671 and *ADH1B* rs1229984 have an active role in alcohol consumption and AUD development. Interestingly, a genome-wide DNA methylation study in Chinese AD patients and siblings without AD as controls indicated that DNA methylation might be an important factor in AD, highlighting the role of *ALDH1L2* as a potential underlying biological mechanism [113]. The same group reported differential methylation between male Chinese AD patients and healthy individuals at multiple CpG sites and observed associations with *ADH1A* [114].

Table 3 summarizes candidate gene studies on AUD that focus on associations between polymorphisms in oxidative stress-related pathways.

### 3.4. GWAS on AUD and Oxidative Stress

GWAS are a useful approach for identifying genetic variants associated with AUD. They can provide important insight, given that AUD is characterized by complex traits, like most psychiatric disorders [13]. However, searching the literature, we could not identify statistically significant associations between AD and oxidative stress-related genes. However, a few studies highlighted the importance of *ADH* and *ALDH* genes, which have also emerged from candidate gene studies mentioned above. It should be noted that most GWAS investigating the genetic influence on AD mainly focus on European populations, leading to an increased risk of missing low-frequency variants [115]. Although limited, there are also published studies that focus on Asian populations.

The first study that provided evidence of a genome-wide significant association in AD was performed in 2009. The findings and statistically significant polymorphisms from rat brains were also replicated in a cohort of 1024 German male patients and 996 matched healthy individuals. *ADH1C* rs1614972 was among the significant polymorphisms replicated in the follow-up study [116]. German Caucasians were also included in a GWAS that indicated the role of rs1789891 on AD. This polymorphism is in LD with *ADH1C* rs1693482 and is located in the ADH gene cluster between *ADH1B* and *ADH1C* [3]. 

A GWAS of self-reported alcohol consumption in a cohort of the UK biobank also verified the association between alcohol metabolizing enzymes and alcohol consumption. Notably, rs145452708, located between *ADH1B* and *ADH1C*, *ADH5* rs29001570, *ADH1C* rs35081954, and rs193099203 found in an intergenic region of 4q23 gave genome-wide significant associations, with the minor alleles associated with decreased alcohol consumption [117]. *ADH1C* rs141973904, previously associated with AUD in cohorts of different ethnicities and populations [3,90], was among the most significant associations of a GWAS that focused on AUDIT scores of 20,328 unrelated European participants [118].

Li et al. performed a GWAS on AD patients and healthy individuals, using two regression models: binary quantitative analyses and polygenic risk scores. They found no genome-wide significant associations. However, rs34361428, located on the *ADH* cluster, was the top associated SNP and was a powerful expression quantitative trait loci (eQTL) for *ADH1B*, *ADH1A,* and both eQTL and splicing quantitative trait loci (sQTL) for *ADH1C* [119].

A GWAS in 2019 included 274,424 participants of multiple ethnicities from the Million Veteran Program and investigated the genetic influence of 686,693 biomarkers on AUDIT-C and AUD. Among the genome-wide significant loci, *ADH1B* rs1229984 and *ADH1C* rs142783062 were associated with AUDIT-C, whereas *ADH1B* rs1229984, *ADH1C* rs1612735, and *ADH4* rs5860563 were associated with AUD [9].

Lai and his colleagues included a cohort of European and African Americans and focused on three phenotypes: DSM-IV AD, DSM-IV AD criterion count, and individual dependence criteria. This trans-ancestral meta-analyses revealed an association between *ADH1B* rs1229984 and AD and DSM-IV AD criterion count. This polymorphism was also associated with the “desire to cut drinking” but only in European Americans [120].

Another GWAS that highlighted the role of the *ADH* cluster in AD included a cohort of European Americans and African Americans and identified population-specific associations. A study including an independent cohort of 1746 European Americans and 803 African Americans replicated the findings. *ADH1B* rs1229984 was statistically significant in European Americans, whereas *ADH1B* rs2066702 and *ADH1C* rs1789882 were in African Americans. Moreover, the authors discovered novel risk loci on chromosome 4 and linked them with the *ADH* gene cluster [85].

The importance of the *ADH1B* locus in alcohol consumption also emerged from a GWAS focused on European and African Americans. The findings showed a significant association between the maximum number of alcoholic beverages participants consumed in the last 24 h. *ADH1B* rs1229984 was significant in European Americans, whereas *ADH1B* rs2066702 was in African Americans [121].

The role of ADH also emerged from a GWAS that included 533 AD patients and 2848 healthy individuals who were Han Chinese males. The authors identified two significant loci, validated in a cohort of 146 AD male patients and 200 healthy male controls. These two significant loci were associated with *ADH1B* rs2075633, *ADH1B* rs1229984, and *ALDH2* rs671. In addition, the influence of these polymorphisms was evaluated on AD characteristics, and it was shown that *ADH1B* rs1229984 was associated with the total score of the Barratt Impulsiveness Scale (BIS-11), the average drinking volume, and the scores of the Michigan Alcoholism Screening Test (MAST), which assesses AD severity [83]. A GWAS that included 1045 subjects of Thai origin also revealed the importance of *ALDH2* rs671 in AD, but no association was observed for *ADH1B* rs1229984. The study assessed three phenotypes, i.e., AD, the maximum number of alcoholic beverages using the Semi-Structured Assessment for Drug Dependence and Alcoholism (SSADDA), and flushing reactions after alcohol consumption. A genome-wide significant association was recorded with variants near *ALDH2* in LD with rs671 [122]. *ALDH2* rs671 also affected alcohol drinker status and the number of drinks consumed per week in the East Asian population. The GWAS also included non-Hispanic whites, in whom *ADH1B* rs1229984 was associated with alcohol intake and the number of drinks per week. This statistically significant result was replicated in Hispanics/Latinos [86]. Finally, the strong association between rs671 and drinking behavior, evaluated using AUDIT, was also observed in residents of Keelung in Taiwan [106].

A study that included Han Chinese patients confirmed the involvement of *ALDH* in alcohol consumption, which has been observed in Europeans. The authors evaluated the association between three AD-related phenotypes and reported statistically significant associations between the *ALDH2* region, flushing response, and the maximum number of drinks in males. The strongest association of the *ALDH2* region was observed for rs671 [115].

Genome-wide significant associations between AD risk and *ADH5*, *ADH4*, *ADH6*, *ADH1A*, *ADH1B*, *ADH7*, and *ALDH2* emerged from a GWAS on a cohort of Korean participants and were replicated in a follow-up study of 504 AD patients and 471 healthy individuals. The replication study included 90 SNPs of the *ADH* cluster, but only *ADH1B* rs1229984 was significantly associated with AD risk [123].

The association of *ALDH2* with drinking behavior has also been observed in the Japanese population in a two-staged genome-wide association study. The GWAS included 456,827 biomarkers, and the significant ones, along with *ADH1B* rs1229984, were replicated in a cohort of 2794 drinkers, 1521 chance drinkers, and 1351 nondrinkers. *ALDH2* rs671 showed the strongest association, and the effect of *ADH1B* rs1229984 was verified [124].

The largest GWAS meta-analysis on AD was performed by the substance use disorders working group of the Psychiatric Genomics Consortium in 2018 and focused on 28 case-control and family studies. It included 14,904 AD patients and 37,944 healthy individuals, stratified by ancestry. Genome-wide significant associations were observed in the ADH gene cluster. *ADH1B* rs1229984 and rs2066702 showed substantial correlations in Europeans and African Americans, respectively. The authors also investigated *ADH1B* expression associations and found that rs1010516440 was a significant eQTL for *ADH1B*, *ADH1A*, and *ADH1C*. rs1010516440 is in LD with rs6827898, and they are located between *ADH1C* and *ADH7*. The protective role of the A allele of rs1010516440 emerged based on its association with increased *ADH1B* expression and lower AD risk. Finally, they observed statistically significant associations between known loci of *ADH1B* and schizophrenia, depression, attention deficit hyperactivity disorder, tobacco, and cannabis [7].

GWAS are considered a powerful tool for the identification of genotype frequency differences between patients and controls, not only because they can analyze a vast number of SNPs across the genome, but also because, unlike candidate gene studies, they do not require prior knowledge regarding genetic risk variants. Similar to candidate gene studies’ findings, GWAS studies also highlight the critical role of polymorphisms in the alcohol metabolism enzyme genes, *ADH* and *ALDH*.

Table 4 summarizes the GWAS on AUD and oxidative stress.

### 3.5. Oxidative Stress, AUD, and DNA Damage

Prolonged excessive alcohol intake contributes to the increased production of ROS that triggers DNA damage [30,125]. An animal study investigated ethanol neurotoxicity through double-stranded RNA-activated protein kinase (PKR), interferon-gamma (IFN-γ; the oxidative stress-inducible regulator of PKR), and its target, p53. It showed that chronic ethanol exposure activates the IFN-γ–PKR–p53 pathway in the frontal cortex of rodents. PKR expression was more significant in the brains of rodents exposed to ethanol at earlier ages than later in life, suggesting a mechanism by which young brains could be more susceptible to ethanol-related brain injury [126]. A clinical study investigating oxidative DNA damage in AD reported increased oxidative DNA damage in patients with AD and its correlation with alcohol withdrawal severity. It compared serum 8-hydroxy-2’-deoxyguanosine (8-OHdG) levels as a marker to estimate ROS-induced DNA damage between patients with AD and healthy controls. They reported that the oxidative DNA damage persisted after 1 week of detoxification and that the alcohol withdrawal severity was correlated with the increase in oxidative stress, particularly the 8-OHdG levels [59]. In a later study, the authors investigated oxidative DNA damage between AD patients with and without delirium tremens, using 8-OHdG as a biomarker in AD patients with and without delirium tremens and reported that AD patients with delirium tremens have higher serum 8-OHdG levels than those without delirium tremens, suggesting that higher oxidative stress carries a greater risk of the occurrence of delirium tremens [60].

A post-mortem analysis of the brain searching for alcohol-responsive genes in the frontal cortex and nucleus accumbens of human alcoholics reported that AD patients expressed downregulation of genes encoding essential proteins involved in vesicular transport cellular architecture in the nucleus accumbens. The authors suggested that the damage may result from oxidative stress, which is known to induce a suite of repair processes [64]. A cell line study confirmed modulation of alcohol on histone deacetylases 2 through oxidative stress mechanisms since a dose-dependent increase in histone deacetylases 2 expressions with ethanol treatment was reported [127].

Recent studies also explored oxidative stress’s possible influence on the telomeres’ length. Telomere shortening is influenced by cumulative exposure to inflammation and oxidative stress and the availability of telomerase, the telomere-lengthening enzyme [128]. A Japanese study investigating the influence of heavy drinking on the onset of age-related diseases by measuring telomere length revealed that telomere length was almost 50% shorter in AD patients than in controls. They also found an association between telomere shortening and thiamine deficiency [58]. As telomeres are triple-guanine-containing sequences that are highly susceptible to damage by oxidative stress, the authors suggested that alcohol metabolism-related ROS generated by CYP2E1 could reduce telomere length by inducing double-strand breaks and interfering with the DNA replication fork [129] and telomere shortening may be accelerated by thiamine deficiency [58]. Another study investigated the influence of psychosocial stress and its impact on telomere length in AD. The study explored whether delayed discounting and childhood trauma is related to leukocyte telomere length in AD patients who are considered to have a higher impulsive choice and shorter telomere length. The authors reported that AD patients and high childhood trauma were significantly related to delay discounting and leukocyte telomere length. In contrast, those with low trauma showed no association [62].

Reports from animal, cell, and human studies show that oxidative DNA damage is associated with alcohol withdrawal severity and delirium tremens. Oxidative DNA damage can influence alcohol-responsive genes in the nucleus accumbens, which is known as the reward center and plays an essential role both in pathogenesis of AUD and remission upon AD treatment. In addition, telomere shortening was found to be associated with prolonged drinking of alcohol, and high childhood trauma can be a confounding factor.

### 3.6. Oxidative Stress and the Pathophysiology of AUD

The pathophysiology of AUD is not clear. BDNF might be related to neuronal plasticity and the reward system [61]. A study investigating BDNF serum and oxidative stress markers in AUD during alcohol detoxification reported that oxidative stress markers serum levels were significantly higher in the AUD patients, while BDNF levels were lower. After alcohol detoxification treatment, the GPX levels in the AUD group dropped, and the BDNF levels rose. The authors suggested that serum BDNF and GPX levels might be state biomarkers for AUD patients undergoing alcohol detoxification [61].

Oxidative stress decreases the expression of the monoamine oxidase A (MAO-A) gene [130]. A rat study investigated how chronic alcohol vapor exposure influences MAO-A activity or level in the prefrontal and anterior cingulate cortex during acute withdrawal. The results showed that chronic ethanol vapor exposure significantly elevated MAO-A activity and protein levels in the prefrontal and anterior cingulate cortex at 24 h withdrawal. The authors suggested a causal relationship between acute alcohol withdrawal and elevated MAO-A levels and activity [131]. Another rodent study investigated monoamine oxidase (MAO) inhibitors (MAOIs) to prevent ethanol-induced brain injury by using KLF11-MAO cell death cascade in the frontal cortex of rats exposed to a modified binge ethanol model and control rats. The results showed that the KLF11-MAO pathway was activated by binge ethanol exposure, and MAOIs were neuroprotective by preventing the binge ethanol-induced changes associated with this cell death cascade [132]. A clinical study investigating the elevation of MAO-A level during AD as the cellular response to oxidative stress and mitochondrial toxicity reported that MAO-A was significantly greater in the prefrontal cortex and all brain regions analyzed in AD. Given the role of MAO-A in oxidative stress, apoptosis, and monoamine metabolism, the authors proposed this abnormality as a therapeutically targetable AD marker. They also reported an association between prefrontal and anterior cingulate cortex MAO-A and severity of depressed mood, which could represent a mechanism to explain dysphoria observed in AD [65]. Animal and human studies confirm a link between oxidative stress and anxiety, depression, and AUD. Oxidative stress might also be involved in the etiology of neuropsychiatric diseases by causing accelerated telomere shortening, mitochondrial dysfunction, inflammation excitotoxicity, and influence on neuronal signaling [23].

Studies suggest that BDNF and GPX levels could be used as biomarkers in AUD patients undergoing alcohol detoxification. The level of MAO-A seems to be influenced by alcohol intake and withdrawal and MAO inhibitors could be used as potential neuroprotective agent to prevent ethanol-induced changes. Since MAO-A is vital in the pathogenesis of depressive and anxiety symptoms, a possible link between oxidative stress and MAO-A could be associated with a higher occurrence of depressive and anxiety disorders in patients with AUD.

### 3.7. Oxidative Stress, Immune System, and Neurodegeneration in AUD

The innate immune gene induced by ethanol and lipopolysaccharides is NOX. This multi-subunit enzyme catalyzes ROS formation, thereby increasing oxidative stress. NOX was first characterized as a phagocytic oxidase in monocytes, where it was hypothesized to contribute to the oxidation of infectious agents. The superoxide produced by NOX can increase NF-κB transcription, thereby creating another amplifying loop of pro-inflammatory signaling [69]. An animal study discovered that lipopolysaccharides and ethanol can increase the expression of NOX subunits, particularly the superoxide-forming gp91phox subunit, in the brain and that ethanol treatment of mice increased superoxide formation in the brain as well as neuronal death. Inhibition of oxidases reduces superoxide formation and protects against alcohol-induced neuronal death [133]. Other studies in mice demonstrated that lipopolysaccharides treatment induced neuroimmune gene expression, NOX activity, and oxidative stress that persisted for at least 20 months and led to neurodegeneration [134]. A cell study showed that ethanol-activated microglial conditioned media enhanced oxidative stress in cultured fetal hypothalamic neuronal cells and increased apoptotic cell death.

Maladaptive changes in oxidative-nitrosative stress signaling have also been reported in AD patients’ frontal cortex of post-mortem brains [133]. A rodent study focused on the contribution of selective prefrontal cortex damage and one-carbon metabolism dysfunction to its alcohol-induced neurological impairments and reported that the prefrontal cortex is more vulnerable to chronic alcohol-induced oxidative stress and neuronal cell death than the hippocampus. This increased vulnerability is evidenced by elevated oxidative stress-induced DNA damage and enhanced expression of apoptotic markers in prefrontal cortex neurons [135]. It was shown that ethanol induces oxidative stress in neurons by increasing the cellular production of ROS, and nitrite, while decreasing the level of the antioxidant glutathione and the cellular activity of antioxidative enzymes (i.e., GPX, CAT, and SOD) [136]. Alcohol-induced activation of microglia results in the microglial release of proinflammatory factors, specifically TNF-α and ROS, which augment neurotoxicity [72]. These findings are consistent with the hypothesis that oxidative stress, by inducing innate immune genes, significantly contributes to alcoholic brain damage and alcoholic neurodegeneration [69]. Oxidative stress markers in alcoholics are considered part of late-stage signs of brain toxicity. A study on young drinkers has demonstrated that oxidative damage biomarkers can be found in individuals with 4–5 years of alcohol drinking history. They displayed reductions in GPX levels and increases in lipid peroxidation, and damaged DNA blood without clinical evidence of hepatic damage [137]. These data strongly implicate oxidative damage in AD’s early and late stages, contributing to brain damage induced by heavy alcohol consumption [50]. Oxidative stress signaling induced by alcohol may not only contribute to cellular injury but also influences the motivational states that drive alcohol consumption [50].

The interplay between oxidative stress, neuroimmune response, and excitotoxicity in AUD leads to neurodegeneration [30]. Chronic alcohol abuse through oxidative reduction response and inflammatory activation leads to cytoskeletal destabilization of BBB integrity, which further activates astrocytes and thus finally causes BBB disruption and neuronal death [68]. Chronic alcohol intake and oxidative stress increase dopamine expression, decrease serotonin expression, and alter the gamma-aminobutyric acid (GABA) A receptor involved in glutamate hyperactivity. Increased dopamine transporter expression increases oxidative stress, and oxidative stress damages dopaminergic neurons [39]. Oxidative stress profoundly affects behavior, similar to what is observed with chronic alcohol intake, indicating that it may be the likely underlying mechanism of alcohol behavioral impairments. Oxidative stress-enhancing activities, PTSD, anxiety, depression, and a personality high in psychoticism, which are states of high brain oxidative stress, when combined with alcohol abuse, which also triggers oxidative stress, may result in the amplification of alcohol-induced impulsive, suicidal, and aggressive behaviors [39].

These above data suggest that NOX plays an important role in ethanol-induced innate immune genes and is associated with neurodegeneration. By inducing innate immune genes, oxidative stress contributes to alcohol-related brain damage and neurodegeneration. Chronic alcohol intake and oxidative stress also influence neurotransmitters and affect behavior and the severity of concurrent mental disorders such as PTSD, anxiety, and depression.

### 3.8. Oxidative Stress, AUD, and Potential Therapeutic Target and Agents

Baclofen, a GABA B agonist, is used to treat AD. A randomized placebo-controlled trial investigated brain metabolites following administration of baclofen in AD individuals. The authors reported significant differences between baclofen and placebo on parietal concentrations of glutathione when controlling for recent drinking. Baclofen-treated participants demonstrated significantly higher glutathione and its neurometabolic ratio than placebo. They conclude that the effect of baclofen may be mediated by increased parietal concentrations of the antioxidant glutathione and N-acetyl aspartate in recently drinking of AD patients [138].

Another potential compound in mitochondrial therapeutics is the structural GABA analog, citrocard (phenibut citrate), which prevents the damaging effect of alcohol and was observed from increased indexes of oxidative phosphorylation in treated animals [139]. Other agents that have received considerable attention as a potential therapeutic against alcohol-induced organ injury are betaine and S-adenosyl-l-methionine, which have beneficial effects on mitochondrial functions. Betaine, also known as trimethylglycine, is a methyl donor which can replace folate or S-adenosyl-l-methionine in the human body [73]. Two rodent studies have demonstrated that the antioxidants, α-lipoic acid and ebselen, significantly reduce alcohol consumption and reinstate alcohol-seeking behavior in rats and mice [140,141].

A rat study explored the effects of NAC on alcohol abstinence and alcohol-induced adverse effects. They investigated the association of NAC intake, alcoholism, and alcohol abstinence on lipid profile, in vivo low-density lipoprotein (LDL) oxidation, oxidative stress, and antioxidant status in the serum and liver of rats. The results showed that ethanol exposure enhanced serum in vivo oxidized-LDL and serum and hepatic oxidative stress [142]. Another rodent study reported that administration of NAC to alcohol-exposed mice significantly reduced symptoms of alcohol withdrawal (i.e., convulsions) [143].

ALDH2 is a promising therapeutic target in numerous diseases and AUD. A functional polymorphism in the *ALDH2* gene and its inactive form causes unpleasant flushing responses after alcohol consumption and prevents excessive alcohol intake [24]. Disulfiram, a potent ALDH2 inhibitor, is an approved drug for the treatment of AUD but has clinical limitations due to the fact of its side effects. An animal study on a mice model reported that liver-targeted ALDH2 inhibition decreased heavy drinking without affecting moderate drinking, which could be a molecular basis for hepatic ALDH2 targeting/editing for the treatment of AUD [144]. Alda-1 [N-(1,3-benzodioxol-5-ylmethyl)-2,6-dichlorobenzamide] is the most studied allosteric ALDH2 agonist [145]. Alda-1 reduces ischemic cardiac damage in other organs, such as the brain, lung, liver, and intestine, and induces neuroprotection; therefore, it might effectively treat AUD [24]. Alda-1 administration reduced the acquisition and the maintenance of ethanol intake in alcohol-preferring rats [146].

Potential therapeutics in AUD include curcumin, a potent scavenger of various ROS, such as superoxide anion, hydroxyl radicals, and nitric oxide [147]. Resveratrol possesses multiple biochemical and physiological actions that include the ability to protect the brain, kidney, and heart from ischemic injury, as it has antioxidant and anti-inflammatory properties [148]. Resveratrol consumption counteracts serum-free oxygen radical formation caused by chronic alcohol intake without influencing the natural, free oxygen radical defense in a mouse model of alcohol addiction. Resveratrol supplementation can also counteract alcohol-induced BDNF elevation in the liver [149]. Pharmacological inhibition of histone deacetylases 2 with trichostatin A may also be of therapeutic significance for treating AUD [127].

Another clinical study examined microvascular dysfunction in an ex vivo experimental model (isolated arterioles) from young adults with repeated binge drinking, moderate alcohol drinking, and alcohol abstention. They tested whether applying the endothelial NOS cofactor tetrahydrobiopterin would restore microvascular function in response to flow and high intraluminal pressure. The results showed that microvascular dysfunction in young adult binge drinkers may be exacerbated with acute pathophysiological stimulus, and that these binge-induced dysfunctions may be reversed by tetrahydrobiopterin [150].

A retrospective clinical study investigated if vitamin K supplementation administered to newborns (to facilitate the synthesis of blood-clotting proteins) might reduce the development of AD later in life. The results showed that vitamin K treatment, inherited risk, and low birth weight independently predicted AD and problem drinking at age 30. Vitamin K treatment was associated with significantly lower AD rates and fewer symptoms of problem drinking, probably by reducing early postnatal hemorrhage and oxidative brain damage [151]. This finding was not replicated in newer studies.

A rodent study investigated whether polyphenols confer a protective potential against alcohol-induced oxidative stress. The results showed that alcoholic mice showed worse oxidative status than nonalcoholic mice, but polyphenol supplementation partially counteracted the alcohol pro-oxidant effects. The authors concluded polyphenols might be dietary-based prevention of the damage associated with chronic alcohol abuse [152].

The increase in plasma carnitine in alcoholic cirrhosis may be related to disordered fatty acid metabolism and oxidative stress in AUD, making carnitine supplementation a treatment for AUD through multiple effects on ethanol metabolism [66].

Cannabidiol (CBD) is a natural component of cannabis that possesses widespread and complex immunomodulatory, antioxidant, anxiolytic, and antiepileptic properties. CBD reduces alcohol-related steatosis and fibrosis in the liver by reducing lipid accumulation, stimulating autophagy, modulating inflammation, reducing oxidative stress, and inducing death of activated hepatic stellate cells. CBD reduces alcohol drinking in animal AUD models by reducing ethanol intake, motivation for ethanol, relapse, anxiety, and impulsivity; it reduces alcohol-related steatosis and liver fibrosis and alcohol-related brain damage [67].

A mice study evaluated treatment with lobeline, a nicotinic receptor antagonist as a candidate for treating alcohol addiction. The anticonvulsant effect of lobeline was assessed using a pilocarpine-induced seizure model. The results showed that lobeline decreased CAT in the hippocampus. Anticonvulsant and neuroprotective actions of lobeline may be mediated by antioxidant-like mechanisms, indicating their potential in alcoholism therapy [153]. A rat study investigated the effect of clavulanic acid on ethanol withdrawal symptoms. Clavulanic acid improved withdrawal-induced anxiety-like behavior and seizure vulnerability induced following ethanol withdrawal. Clavulanic acid attenuated also increased MDA and decreased glutathione after withdrawal. The authors concluded that alcohol withdrawal causes oxidative stress, which can be prevented by clavulanic acid [154].

Many potential therapeutic agents and antioxidants were proposed to decrease oxidative stress in AUD, but more extensive human studies are needed to confirm these findings. Table 5 presents an overview and more details on published animal studies, while Table 6 summarizes published human studies on alcohol use disorder and oxidative stress with potential therapeutic agents.

### 3.9. Registered Clinical Trials on Potential Therapeutic Targets and Agents in AUD-Related Oxidative Stress

Most of the registered clinical trials investigated the effect of N-acetylcysteine (NAC) on AUD. Some of them combine the impact of NAC with other drugs, such as topiramate (TPM), gabapentin, naltrexone, and cognitive behavioral therapy. So far, only four trials have been completed and reported results. One of them reported no statistically significant results between the groups of placebo and low and high doses of NAC. The alcohol units did not differ among these three groups. The placebo group had 3.1 (3.5), whereas the groups of low- and high-NAC doses had 2.8 (3.5) and 3.8 (3.6) alcohol units, respectively (NCT03216954). A study on veterans that evaluated the change in the percent of heavy drinking days per week assessed by the timeline follow back (TFLB) method did not find significant differences among the investigated groups. The change in the percent of heavy drinking days per week for the NAC group was 26.2 (0.31), while for the placebo, it was 26.5 (0.39) (NCT02791945).

The impact of NAC in reducing alcohol drinking and craving was evaluated in two groups: one receiving NAC and one placebo. Initially, the percentage of heavy drinking days in the intervention group that received NAC at week 1 was 70.2 (7.1), and at week 9 it was 20.2 (8.6). While in the placebo group, the percentage was 58.4 (6.7) at week 1 and 14.7 (5.5) at week 9 (NCT00568087).

A study using a combination of NAC with a high dose of naltrexone (150 mg) compared the combination group against two groups of patients receiving a high dose of naltrexone and a low dose of naltrexone (50 mg) alone, evaluating the percentage of heavy drinking days, measured using the timeline follow back (TLFB) method, over 12 weeks. The percentage of heavy drinking days at week 1 was 45.0 (4.5), 50.6 (4.3), and 44.0 (4.5) for patients receiving the combination of NAC and high dose of naltrexone, high dose, and low dose of naltrexone, respectively. At week 13, a reduction in the percentage was recorded for all three groups (i.e., combination of NAC and high dose of naltrexone: 5.2 (5.6); high dose of naltrexone 3.4 (5.1); and low dose of naltrexone 3.3 (5.3)) (NCT01214083).

Three studies included AUD patients with concurrent bipolar disorder, traumatic brain injury (TBI), cocaine addiction, and PTSD. The findings are reported only from the latter, indicating that AUD and PTSD severity and alcohol craving improve after treatment with NAC.

In addition, several studies evaluated the effect of herbal dietary supplements as antioxidant therapy, such as livitol-70, on liver function and Protandim in patients with alcohol abuse. The impact of the diabetes drug, pioglitazone, on the alcoholic lung was also suggested. However, the study was terminated due to the COVID-19 pandemic. Finally, one study evaluated the effect of melatonin in AD patients with sleep problems.

To sum up, the complete registered clinical trials with available results lead to conflicting findings regarding the effect of NAC treatment in patients with AUD. Two of them reported no statistically significant results (i.e., NTC03216954 and NTC02791945), whereas another study provided evidence of a reduction in heavy drinking days but for both the group treated with NAC and the placebo (i.e., NTC00568087). The same results emerged when the combination of NAC and a high or low dose of naltrexone was administered to AUD patients (i.e., NCT01214083). Based on the above, it remains unclear whether interventions such as NAC can have a protective role for AUD, and more studies are needed to shed light on the underlying mechanisms.

Table 7 summarizes the relevant clinical trials registered on ClinicalTrials.gov.

## 4. Conclusions

A huge body of data indicate that oxidative stress is detrimental to the etiology of AUD, both as a factor contributing to increased alcohol consumption as well as the pathophysiology of acute and chronic alcohol toxicity. Chronic alcohol consumption leads to increased oxidative stress, and the imbalance between the production of free radicals and the antioxidant systems, such as SOD, CAT, and GPX, triggers lipid peroxidation, protein inactivation, and DNA damage leading to mitochondrial dysfunction, cell death, and increased inflammatory response. The brain is especially vulnerable to the effects of alcohol, as it rapidly diffuses through the blood–brain barrier and alters neurotransmission. Furthermore, processes that are detrimental to the proper functioning of the brain may be affected due to the increased oxidative stress-related damage and neuroinflammation, leading to altered neuronal signaling and inhibition of neurogenesis.

We have identified several preclinical studies in animal models and clinical trials investigating potential therapeutic targets and agents that could reduce the severity of AUD and protect from the damaging effects of alcohol or counteract them. However, it is important to keep in mind that pathways implicated in alcohol metabolism, oxidative stress defense systems, inflammation, and neurodegeneration are under genetic control. Due to the genetic variability, one therapeutic approach may not fit all patients. Better understanding of underlying genetic factors regulating alcohol-related pathways may be the key to personalized treatment of AUD.

## Figures and Tables

**Table 1:** Overview of clinical studies on AUD and oxidative stress.

Topic of the Study	Aim of the Study	Number of Patients Included	Significant Findings, Safety, Disease Response, and Disease Control	Type of Study	Reference
Evaluation of oxidative stress biomarkers, liver, and renal function parameters in patients during AD treatment	To compare oxidative stress and renal and hepatic function parameters upon admission and discharge from the hospital	28	Chlorpromazine showed influence over hepatic function markers and oxidative stress parameters (i.e., CAT and GPX); carbamazepine influenced hepatic function and ferric reducing antioxidant power; SOD levels were lower, and GPX and ferric reducing antioxidant power presented higher levels at discharge	Prospective cohort study	[51]
Influence of heavy drinking on the onset of age-related diseases by measuring telomere length	To measure telomere length of Japanese patients with AD and search for an association between telomere length and genetic variants of *ADH1B* and *ALDH2*	255	Telomere length was almost 50% shorter in AD patients relative to the controls. There were no associations between *ADH1B* and *ALDH2* genotypes and telomere length	Cohort study	[58]
The correlation between early alcohol withdrawal severity and oxidative stress in AD patients	To explore the correlation between alcohol withdrawal severity and two oxidative stress markers: MDA and SOD	95	Compared to the controls, serum MDA levels were significantly elevated, and SOD activity was significantly lowered in alcoholic patients; clinical withdrawal severity was significantly positively correlated with serum MDA levels	Cohort study	[55]
Increased oxidative DNA damage in AD patients and its correlation with alcohol withdrawal severity	To compare serum 8-OHdG levels between patients with AD and healthy controls and to investigate the correlation between this marker and the severity of alcohol withdrawal syndrome	142	The oxidative DNA damage persisted after 1 week of detoxification. The alcohol withdrawal syndrome severity was correlated with the increase in oxidative stress	Prospective cohort study	[59]
Comparison of oxidative DNA damage between AD patients with and without delirium tremens	To investigate levels of 8-hydroxy-2’-deoxyguanosine (8-OhdG) as a marker of oxidative DNA damage in AD patients	74	AD patients with delirium tremens had higher serum 8-OhdG levels than those without delirium tremens, suggesting that higher oxidative stress carries a greater risk of the occurrence of delirium tremens	Prospective cohort study	[60]
BDNF and GPX as state biomarkers in AUD patients undergoing detoxification	To investigate the serum levels of BDNF and oxidative stress markers in AUD patients during alcohol detoxification	34	Serum levels of oxidative stress markers were significantly higher in the AUD group than in control group, while BDNF levels were lower; after alcohol detoxification treatment, the GPX levels in the AUD group dropped, and the BDNF levels rose	Cohort study	[61]
Telomere length in AD and its role in impulsive choice and childhood maltreatment	To examine whether delayed discounting and childhood trauma are related to leukocyte telomere length in AD patients, who are considered to have a higher impulsive choice and shorter telomere length	253	Patients with AD and high childhood trauma showed a significant relationship between delay discounting and leukocyte telomere length, while those with low trauma showed no association between them	Prospective study	[62]
Alterations in oxidative stress status during early alcohol withdrawal in alcoholic patients	To investigate serial alterations in various oxidative stress markers during early detoxification in alcoholic patients	140	Marked oxidative stress in alcoholic patients without severe liver disease was observed; the attenuation of a raised MDA level and lowering of CAT activity appeared after one week of detoxification; alcoholic patients did not scavenge free radicals as readily as controls	Prospective cohort study	[22]
Oxidative damage to plasma proteins in patients with chronic AD and the effect of smoking	To examine the oxidative status of plasma proteins as markers of oxidative stress in subjects with chronic AD with smoking as a cofounding factor	132	Systemic oxidative stress in chronic AD was attributed mainly to alcohol consumption, while smoking may act synergistically	Prospective cohort study	[56]
Relationship between liver function and brain shrinkage in AD patients	To assess the correlations between liver function and brain volume measurements in AD patients	235	The results showed that higher liver function levels correlated with brain volume shrinkage in AD patients but not in the controls. Serum gamma-glutamyl transferase levels outweighed the aging effect on brain shrinkage in female patients	Prospective cohort study	[63]
Oxidative status in AD patients	To examine the relationship between AD and oxidative status	47	Serum MDA levels of AD patients were found to be significantly increased compared with the controls and decreased after abstinence; serum CAT did not return to normal status at week 2 after abstinence; the activity of CAT was significantly correlated with MDA levels	Prospective cohort study	[54]
Oxidoreductive homeostasis in AD male patients and the risk of alcohol drinking relapse in a 6 month follow up	To verify the hypothesis that oxidoreductive blood balance can also affect demand for energy substances, such as alcoholic beverages, in AD individuals as well as the severity of their AD and risk of drinking relapse	77	The risk of alcohol drinking relapse was lower in patients with an above-median initial blood concentration of nitric oxide metabolites and total antioxidant status; the oxidative stress parameters correlated with AD severity markers	Prospective cohort study	[57]
Alcohol-responsive genes in the frontal cortex and nucleus accumbens of human alcoholics	To compare the RNA expression profile of the nucleus accumbens and prefrontal cortex of the human brain from matched individual alcoholic and control cases	14	Downregulation of genes encoding essential proteins involved in vesicular transport and cellular architecture in nucleus accumbens of the alcoholic	Comparative postmortem study	[64]
MAO-A levels in brain regions in AD	To verify the hypothesis that the MAO-A level is elevated in the prefrontal cortex during AD as the cellular response to oxidative stress and mitochondrial toxicity	32	MAO-A was significantly greater in the prefrontal cortex and all brain regions analyzed in AD; an association between prefrontal and anterior cingulate cortex MAO-A and the severity of depressed mood was observed	Cohort study	[65]
Investigation of antioxidant activity of human serum	To conduct a comparative investigation of the total antioxidant activity of human serum	30	All applied methods revealed that the serum total antioxidant activity of the AD patients was lower than the total antioxidant activity of the control group	Cohort study	[52]
Concentrations of manganese SOD in the serum of AD patients	The quantitative determination of the plasma concentrations of oxidative stress-associated parameters (concentrations of lactoferrin, Cu, Zn-SOD, and Mn-SOD) in AD and controls	35	Increased oxidative stress was observed in AD patients	Cohort study	[53]

Alcohol dependence (AD); catalase (CAT); glutathione peroxidase (GPX); superoxide dismutase (SOD); malondialdehyde (MDA); monoamine oxidase A (MAO-A).

**Table 2:** Review articles from which additional information on AUD and oxidative stress was extracted.

Aim of the Review	Significant Findings	Reference
To review principles in alcohol-induced neurodegeneration; the interplay between oxidative stress, neuroimmune response, and excitotoxicity	Alcohol-induced oxidative stress initiated the innate immune response; no direct link between an alcohol-induced hyperglutamatergic state and excitotoxicity in humans; neuroimmune response and excitotoxicity leading to neurodegeneration (i.e., apoptosis and necrosis); chronic alcohol intake has the potential for the development of neurodegenerative diseases; the interplay between oxidative stress, neuroimmune response, and excitotoxicity leading to neurodegeneration	[30]
To review *ALDH2* polymorphism in disease, aging, alcohol addiction, and its potential as a therapeutic target	*ALDH2* plays a vital role in the pathogenesis of human conditions such as AUD, cancer, cardiovascular diseases, diabetes mellitus, and neurodegenerative diseases; the clearance of endogenous aldehydes mediates its effect; animal model studies suggested an *ALDH2* activator, Alda-1, may have a preventive effect against neurodegenerative diseases including AUD	[24]
To review the neurobiological basis for alcohol-induced aggression, impulsivity, and suicidal behavior	Oxidative stress plays a critical underlying role in alcohol toxicity and behavioral impairments; antioxidant therapy should be an integral part of acute alcohol intoxication and AUD treatment	[39]
To review connections between carnitine metabolism and the pathophysiology of the AUD	Alcohol use appears to impact carnitine metabolism, most clearly in the setting of alcoholic cirrhosis; an increase in plasma carnitine may be related to disordered fatty acid metabolism and oxidative stress in AUD; carnitine can be a supplementation in the treatment of AUD	[66]
To provide a rationale for using CBD to treat human subjects with AUD, based on the findings of experimental studies	CBD reduces alcohol-related steatosis and fibrosis in the liver by reducing lipid accumulation, stimulating autophagy, modulating inflammation, reducing oxidative stress, and inducing death of activated hepatic stellate cells; CBD reduces the level of alcohol drinking in animal models of AUD by reducing ethanol intake, motivation for ethanol, relapse, anxiety, and impulsivity; it reduces alcohol-related steatosis and fibrosis in the liver and reduces alcohol-related brain damage	[67]
To review the mechanisms of alcohol on the pathological relationships of neurodegeneration that cause permanent neuronal damage in AUD	Chronic alcohol abuse through oxidative reduction response and inflammatory activation leads to cytoskeletal destabilization of BBB integrity, which further activates astrocytes and, thus, finally causes BBB disruption and neuronal death	[68]
To review if anxiety disorders, depression, and AUD share oxidative stress in their etiologies	Animal and human studies confirm a link between oxidative stress and anxiety, depression, and AUD. Oxidative stress might also be involved in the etiology of neuropsychiatric diseases by causing accelerated telomere shortening, mitochondrial dysfunction, inflammation excitotoxicity, and influence neuronal signaling	[23]
To review how induction of neuroimmune genes by binge drinking increases neuronal excitability and oxidative stress, contributing to the neurobiology of AD	Ethanol-induced immune gene, NOX, catalyzes the formation of ROS and superoxide and thereby increases oxidative stress; oxidative stress, by inducing innate immune genes, significantly contributes to alcoholic brain damage and alcoholic neurodegeneration	[69]
To review the interrelationship between H_2_S signaling and cigarette smoking or alcohol drinking	The evidence from cellular and animal studies and also clinical observations identify H_2_S as a regulator of oxidative stress and inflammatory response in the pathogenesis of various diseases associated with cigarette smoking and alcohol drinking	[70]
To review neuroimmune factors, such as cytokines, Toll-like receptors (TLRs), and HMGB1, and the neuroimmune signaling influence of alcohol drinking habits	The findings support the hypothesis that adolescence is a period of risk for persistent and long-lasting increases in brain neuroimmune gene expression that promote persistent and long-term increases in alcohol consumption, neuroimmune gene induction, and neurodegeneration associated with AUD	[71]
To review the role of microglia in the regulation of ethanol neurotoxic action	Microglia, the immune cells of the central nervous system, play an essential role in modulating alcohol-induced neurotoxicity. Microglia are implicated in alcohol-induced neuroinflammation and alcohol-induced increases in oxidative stress	[72]
To investigate how alcohol abuse causes damage to and functional impairment of organs, such as the heart, stomach, liver, and nervous system, and its prenatal effects	The potential target of compounds that can be used to prevent therapies for alcohol abusers are listed (e.g., curcumin, resveratrol, piceatannol, *S*-adenosyl-l-methionine, and betaine)	[73]
To evaluate the role of glutathione and redox signaling in cocaine, methamphetamine, and alcohol addiction	Redox signaling through oxidation and reduction reactions plays an essential role in numerous cell-signaling cascades including those with opposing cellular consequences, proliferation, and apoptosis; oxidative damage in the early and late stages of AD is a contributing factor to brain damage induced by heavy alcohol consumption; chronic alcohol exposure in humans and rodents can decrease glutathione and glutathione reductase levels in the brain and alter GST in the brain, blood, and saliva; oxidative stress signaling induced by alcohol may not only contribute to cellular injury but also influences the motivational states that drive alcohol consumption	[50]

Blood–brain barrier (BBB); glutathione S-transferase (GST); reactive oxygen species (ROS); NADPH oxidase (NOX).

**Table 3:** Candidate gene studies in AUD that focused on associations with polymorphisms in oxidative stress-related pathways.

Genes and Genetic Variations Studied	Number of Subjects	Ethnicity	Genotyping Method	Outcomes	Reference
*CAT* rs1001179	201 patients and 97 controls	Slovenian	TaqMan	Association between rs1001179 and AD and AUDIT scores	[89]
*CAT* rs1001179	85	African American	TaqMan	No association	[28]
*GSTP1* rs1695	39 patients and 43 controls	Central Brazilians	Allele-specific PCR and sequencing	Association between rs1695 and AUD	[21]
*GSTM1* (*1, *2), *GSTT1* (*1, *2)	200 patients and 400 controls	North Italian	PCR-RFLP	Association between the *GSTM1* null genotype and ethanol intake	[90]
*ADH1B* (*1, *2), *ALDH2* (*1, *2), GSTM1 (*1, *2), *GSTT1* (*1, *2),	121 patients and 145 controls	Central Indians	PCR-RFLP	Association between ALDH*2/*2 and the GSTT1 null genotype with alcohol consumption	[11]
*ADH2* (*1, *2, *3), *ADH3* (*1, *2), *ALDH2* (*1, *2)	80 AD patients and 144 controls	Han Chinese	PCR-RFLP	Protective role of ALDH*2/*2 against AD	[107]
*ADH1B* rs122994, *ALDH2* rs671	34 AD patients and 121 controls	Japanese	PCR-RFLP	Associations between *ADH1B* rs122994, *ALDH2* rs671, and AD	[58]
9 polymorphisms in *ALDH2* and 41 in *ADH*	4597	Northern European	Sequenom mass array	Association between *ADH1B* rs1042026 and alcohol intake; an association of *ADH1C* rs1693482 and *ADH5* rs1230165 with alcohol consumption	[91]
*ADH1B* (*1, *2)	152	Jewish American	Enzymatic amplification followed by hybridization with allele-specific oligonucleotides	Association between ADH1B*2 and lower rates of alcohol consumption and more unpleasant reactions	[95]
*ADH1A* rs2276332, *ADH1B* rs1229984 and rs4147536, *ADH1C* rs11499823, rs4147541, and rs1693431, *ALDH2* rs671, rs4646778, rs886205, rs4646775	5451	Japanese	TaqMan SNP Genotyping Assays	Significant association between *ADH1B* rs1229984 and *ALDH2* rs671 with drinking behavior	[93]
*ADH2* (*1, *2, *3), *ADH3* (*1, *2), *ALDH2* (*1, *2)	340	Mission Indian	Allele-specific PCR	Association of ADH2*1 with AD and ADH2*3 with AD and a lower number of maximum drinks	[99]
ALDH1A1*2 and ALDH1A1*3	137 AD patients and 108 controls	Trinidadian and Toboggan (either Africans or Indians)	Sequencing	Association of ALDH1A1*2 with a lifetime diagnosis of AD and high levels of alcohol consumption	[104]
175 SNPs in 17 alcohol-metabolizing genes	1588	European American	NA	Strong association between *ADH1B* rs1229984 and the DSM-IV symptom count and the maximum number of drinks	[94]
*ADH2* (*1, *2), *ADH3* (*1, *2), *ALDH2* (*1, *2)	34 AD patients and 92 controls	New Zealand (White, Asian, and Polynesian (New Zealand Maori and others))	A newly developed DNA sequencing assay	Protective role of ADH2*2 allele against AUD	[100]
110 SNPs in *ADH7*, *ADH1C*, *ADH1B*, *ADH1A*, *ADH6*, *ADH4*, *ADH5*	NA	European American and African American	Sequenom mass array system	Significant associations between *ADH4* rs1984362, rs4699718, rs3762894, rs4148886, rs4699714, rs7694646, rs1126672, DWSHpy188I, rs1042364, rs1042365, rs2602866, and rs2602846 and AUD	[101]
103 SNPs in the *ADH* gene region	812	Northern and Southern European	Sequenom mass array system	Associations between *ADH1B* rs2018417, rs1229985, rs17033, and rs1789877; *ADH1A* rs931635, rs1229967, rs1618572, rs1230025, and rs2276332; rs3857224, and rs3762894 in the *ADH6*–*ADH4* intergenic region and *ADH4* rs1800759 with early and late stages of alcohol metabolism	[97]
*ALDH2* (*1, *2)	68 AUD patients and 232 controls	Korean	PCR-RFLP	Association between ALDH2*1 and AUD	[105]
*ADH1B* rs1159918, *ADH1C* rs1614972, *ADH4* rs1042364, *ALDH2* rs2238151	99 AUD patients and 100 controls	Central-West Brazilians	TaqMan assay	Statistically significant association between *ADH4* rs1042364, *ALDH2* rs2238151, and AUD	[102]
*ADH2* (*1, *2), *ALDH2* (*1, *2)	153 AUD patients and 153 controls	Japanese	PCR-RFLP	Association between ALDH2*1/*1 and ADH2*1/*1 with AUD	[98]
*ALDH1A1* rs348479, rs348472, rs610529, rs2288087, rs13959, rs348449, rs595958, rs918836	104 alcoholics and 201 controls	Finnish	TaqMan	Association between *ALDH1A1* rs348479 and rs610529 with AD	[103]
~750,000 genomic variants	71 heavy drinkers and 126 controls	Taiwanese	High-density SNP arrays	Strong association between *ALDH2* rs671 and drinking behavior	[106]
*ADH1B* rs1229984	5632	European and African American	KASPar assays or Sequenom	Protective effect of *ADH1B* rs1229984 against AD	[92]
43 SNPs	808 AD patients and 1248 controls	European	Illumina 660 genome-wide SNP array, Illumina BeadXpress platform using a VeraCode SNP panel	Association between *ADH1C* rs1614972 and AD	[96]

Not available (NA); alcohol dependence (AD); alcohol use disorder (AUD).

**Table 4:** GWAS on AUD that observed an association with ethanol metabolism or oxidative stress genes.

Outcome	Number of Subjects	Ethnicity	Platform	References
Association between *ADH1C* rs1614972 and AD	487 AD male patients and 1358 controls	Caucasian (German)	Human-Hap 550 BeadChip	[116]
Genome-wide significant association between alcohol intake and the number of drinks per week and *ALDH2* rs671 in East Asians and *ADH1B* rs1229984 in non-Hispanic whites and Hispanic/Latino	86,627	Non-Hispanic Whites, Hispanic/Latinos, East Asians, and African Americans	Affymetrix Axiom arrays	[86]
Genome-wide significant associations between AD risk and *ADH5, ADH4, ADH6, ADH1A, ADH1B, ADH7,* and *ALDH2*	117 AD patients and 279 controls	Korean	Illumina Human660 W BeadChip	[123]
Genome-wide significant associations between *ADH1B* rs1229984 and AD and DSM-IV AD criterion count	7418 (1121 families), 3175 (585 families)	European American and African American	Illumina Human1M array, Illumina Human OmniExpress 12V1 array, Illumina 2.5M array, Smokescreen genotyping array	[120]
Genome-wide significant associations with variants near *ALDH2*	1045	Thai	Illumina Global Screening Array (GSA) and Illumina Multi-Ethnic Global Array (MEGA)	[122]
Identification of two significant loci that were associated with *ADH1B* rs2075633, *ADH1B* rs1229984, and *ALDH2* rs671	533 males with AD and 2848 controls	Han Chinese	Illumina Global Screening Array-24 v1.0 BeadChip	[83]
Association between rs1229978 (near *ADH1C*) with AUDIT-C, and rs1154433 (near *ADH1C*) and *ADH4* rs5860563 with AUD	274,424	European American, African American, Latino American, East, and South Asian American	Affymetrix Axiom Biobank Array	[9]
Association between *ADH1C* rs141973904 and AUD	20,328	European	Illumina HumanHap550+ BeadChip V1 V2, OmniExpress + BeadChip V3, Custom array V4	[118]
Association between rs1789891 and AD	1333 male patients with severe AD and 2168 controls	Caucasian (German)	Illumina Human610Quad or 660w Quad BeadChip (patients), Illumina HumanHap550 BeadChip (controls)	[3]
Association between rs34361428 and AD	739 patients with ADS and 251 controls	English, Scottish, Welsh, or Irish	Illumina PsychArray	[119]
Association between AD and *ADH1B* rs1229984 in European Americans, and *ADH1B* rs2066702 and *ADH1C* rs1789882 in African Americans	5697	European American and African American	Illumina HumanOmni1-Quad v1.0 microarray	[85]
Association between *ALDH2* rs671 and AD, flushing response, and maximum drinks in males	313	Han Chinese	Illumina Cyto12 array version 2-1	[115]
Genome-wide significant associations between alcohol consumption and *ADH5* rs29001570, *ADH1C* rs35081954, and rs145452708 located in the region between *ADH1B* and *ADH1C*, and rs193099203 located in an 4q23 intergenic region	112,117	White British	Affymetrix UK Biobank Axiom array, Affymetrix UK BiLEVE Axiom array	[117]
Association between alcohol consumption and *ALDH2* rs671 and *ADH1B* rs1229984	733 cases and 729 controls	Japanese	Infinium HumanHap550 Bead Array (Illumina)	[124]
Strong association between *ALDH2* rs671 and drinking behavior, evaluated using AUDIT	71 heavy drinkers and 126 controls	Taiwanese	Affymetrix Axiom Genome-Wide TWB 2.0 array	[106]
Significant associations between the maximum number of drinks and *ADH1B* rs1229984 in European Americans and *ADH1B* rs2066702 in African Americans	9500	European American and African American	Illumina HumanOmnil-Quad v1.0 microarray	[121]

Alcohol dependence syndrome (ADS); alcohol use disorder (AUD); alcohol dependence (AD).

**Table 5:** Preclinical studies of potential therapeutic agents in animal models.

Topic of the Study	Aim of the Study	Number of Animals Included	Significant Findings, Safety, Disease Response, and Disease Control	Type of Study	Reference
Oxidative stress inhibition by resveratrol in AD mice	Administration of different dosages of resveratrol in alcoholic adult male mice and measuring oxygen radical levels and alteration of BDNF in the liver	5	Prolonged resveratrol consumption counteracts serum-free oxygen radical formation caused by chronic alcohol intake without influencing the natural free oxygen radical defense in a mouse model of alcohol addiction Resveratrol supplementation can counteract alcohol-induced BDNF elevation in the liver	Prospective animal study	[149]
Chronic voluntary alcohol drinking and anxiety-like behavior, thiamine deficiency, and brain damage in alcohol-preferring mice	To evaluate the effects of alcohol on neurobehavioral and neuropathological changes in a mouse model	7	Chronic voluntary drinking caused anxiety-like behaviors; alcohol increased the expression of neuroinflammation markers and caspase-3 and glial fibrillary acidic protein; alcohol inhibited the expression of thiamine transporters in the brain and reduced thiamine levels in the blood and caused oxidative stress and ER stress and stimulated neurogenesis	Prospective animal study	[48]
Lobeline as a potential treatment for drug abuse	To evaluate the possible anticonvulsant and neuroprotective activities of lobeline as a candidate in the treatment of alcohol addiction	69	Lobeline decreased CAT in the hippocampus; lobeline has anticonvulsant and neuroprotective actions that may be mediated by antioxidant-like mechanisms, indicating its potential as a candidate drug in alcoholism therapy	Prospective animal study	[153]
Effects of NAC on alcohol abstinence and alcohol-induced adverse effects in rats	To investigate the association of NAC intake, alcoholism, and alcohol abstinence on lipid profile, in vivo LDL oxidation, oxidative stress, and antioxidant status in the serum and liver of rats	30	Ethanol exposure enhanced serum in vivo oxidized-LDL as well as serum and hepatic oxidative stress	Prospective animal study	[142]
Potential role of clavulanic acid in ethanol withdrawal	To investigate the effect of clavulanic acid on the symptoms of ethanol withdrawal in rats	126	Clavulanic acid improved withdrawal-induced anxiety-like behavior and seizure vulnerability induced following ethanol withdrawal	Prospective animal study	[154]
Level of monoamine oxidase A activity and protein levels in rodent brain during acute withdrawal after chronic intermittent ethanol vapor exposure	To determine whether chronic alcohol vapor exposure causes upregulation of MAO-A activity or levels in the prefrontal and anterior cingulate cortex of rodents during acute withdrawal	16	Chronic ethanol vapor exposure significantly elevated MAO-A activity and protein levels in the prefrontal and anterior cingulate cortex at 24 h withdrawal	Prospective animal study	[131]
Olive polyphenol’s effects in a mouse model of chronic ethanol addiction	To determine whether polyphenols confer a protective potential against alcohol-induced oxidative stress	40	Alcoholic mice showed a worse oxidative status than nonalcoholic mice, but polyphenol supplementation partially counteracted the alcohol pro-oxidant effects	Prospective animal study	[152]
Ethanol-mediated upregulation of interferon-gamma, double-stranded RNA-activated protein kinase and p53	To investigate the upregulation of the double-stranded RNA-activated protein kinase signaling pathway by ethanol	54	Chronic ethanol exposure activates the IFN-γ–PKR–p53 pathway in the frontal cortex of rodents. Double-stranded RNA-activated protein kinase expression was more significant in the brains of rodents exposed to ethanol at earlier ages compared to later in life, suggesting a mechanism by which young brains could be more susceptible to ethanol-related brain injury	Prospective animal study	[126]
Sensitivity of the prefrontal cortex and hippocampus to alcohol-induced toxicity	To gain a better understanding of the potential contribution of selective prefrontal cortex damage and one-carbon metabolism dysfunction to its alcohol-induced neurological impairments	10	The prefrontal cortex is more vulnerable to chronic alcohol-induced oxidative stress and neuronal cell death than the hippocampus	Prospective animal study	[135]
Genetic vulnerability to alcohol withdrawal and brain mitochondrial oxidative homeostasis	To elucidate the mechanisms involved in the actions of a QTL with a significant effect on genetic predisposition to alcohol withdrawal	54	Administration of NAC significantly reduces symptoms of alcohol withdrawal (i.e., convulsions) in mice	Prospective animal study	[143]
Examining the use of MAO inhibitors to prevent ethanol-induced brain injury	To investigate the ethanol-mediated KLF11-MAO cell death cascade in the frontal cortex of rats exposed to a modified binge ethanol model and control rats	64	The KLF11-MAO pathway is activated by binge ethanol exposure, and MAOIs are neuroprotective by preventing the binge ethanol-induced changes associated with this cell death cascade	Prospective animal study	[132]

Brain-derived neurotrophic factors (BDNFs); endoplasmic reticulum (ER); low-density lipoprotein (LDL); quantitative trait loci (QTL); monoamine oxidase inhibitors (MAOIs); N-acetylcysteine (NAC).

**Table 6:** Published clinical studies on potential therapeutic agents in humans.

Topic of the Study	Aim of the Study	Number of Patients Included	Significant Findings, Safety, Disease Response, and Disease Control	Type of Study	Reference
Baclofen in AD treatment	To examine brain metabolites following administration of baclofen or placebo in AD individuals	31	There were significant differences between baclofen and placebo on parietal concentrations of glutathione when controlling for recent drinking, with baclofen-treated participants demonstrating significantly higher levels of GSH/Cr ratios relative to placebo	Randomized placebo-controlled trial	[138]
Influence of neonatal vitamin K and vulnerability to AD	To test the hypothesis that vitamin K supplementation administered to newborns facilitates the synthesis of blood-clotting proteins that might reduce the development of AD later in life	238	Vitamin K treatment was associated with significantly lower rates of AD and fewer symptoms of problem drinking	Retrospective cohort study	[151]
Tetrahydrobiopterin and microvascular dysfunction in young adult binge drinkers	To examine microvascular dysfunction in an ex vivo experimental model (isolated arterioles) from young adults with a history of repeated binge drinking, moderate alcohol drinking, and alcohol abstention and the role of tetrahydrobiopterin	36	In young adult binge drinkers, microvascular dysfunction may be exacerbated with acute pathophysiological stimulus; these binge-induced dysfunctions may be reversed by tetrahydrobiopterin	Cohort study	[150]

Alcohol dependence (AD).

**Table 7:** Registered clinical trials.

Study Title	NCT Identifier	Number of Patients	Condition	Intervention	Endpoints	Study Status	Results
NAC for Adolescent AUD	NCT03707951	120	AUD	NAC, placebo	Effects of NAC on a platform of weekly evidence-based brief alcohol intervention	Recruiting	NA
A Study of NAC for AUD	NCT04964843	50	AUD	NAC, placebo	Assess the impact of NAC on AUD	Not yet recruiting	NA
Influence of NAC Maintenance on Alcohol Effects Completed	NCT03216954	14	AUD	Alcohol, placebo, NAC	Evaluate the behavioral effects of alcohol during placebo and NAC maintenance	Complete	No statistically significant results
Topiramate Augmenting Strategies for the Treatment of AUD	NCT03120468	16	Alcoholism	TPM and NAC, TPM and placebo	Evaluate the safety and tolerability of TPM + NAC versus TPM + placebo for AUD treatment	Active, not recruiting	NA
Clinical Trial for AUD and PTSD	NCT02966873	200	Addiction, alcohol abuse	NAC, placebo, cognitive behavioral therapy	Evaluate the effects of NAC in reducing AUD severity and PTSD symptomatology	Active, not recruiting	NA
Melatonin Use for Sleep Problems in AD Patients	NCT03043443	60	Alcohol-related disorders	Melatonin, placebo	Record sleeping problems	Completed	NA
NAC Treatment of AUD In Veterans With TBI	NCT02791945	30	TBI, hazardous and harmful alcohol use	Medical management counseling, NAC, placebo	Assess the efficacy of NAC in reducing alcohol use and improving brain injury symptoms in veterans with mTBI who consume alcohol at hazardous or harmful levels	Completed	No statistically significant results
Imaging GABAergic/Glutamatergic Drugs in Bipolar Alcoholics	NCT03220776	81	AUD, BD	NAC, gabapentin, placebo	Manipulate neurochemical Dysfunctions characteristic of individuals with co-occurring BD and AUD, using gabapentin and NAC, and evaluate medication-related changes in response inhibition and alcohol cue-reactivity fMRI tasks, as well as drinking and mood in individuals with AUD + BD	Recruiting	NA
NAC for Treating Comorbid PTSD and SUD	NCT02911285	90	PTSD, AUD, SUD	NAC, placebo, cognitive behavioral therapy	Determine the benefits of NAC in treating AUD and comorbid PTSD	Completed	Changes in AUD and PTSD severity and alcohol craving
Herbal Supplements for Improvement of Liver Function in Participants with Alcoholic Liver Disease	NCT03503708	40	Alcoholic liver disease	Livitol-70	Study the efficacy of the herbal supplement to improve liver functioning of alcoholic liver disease patients	Not yet recruiting	NA
Effect of NAC on Alcohol and Cocaine Use Disorders: A Double-Blind Randomized Controlled Trial	NCT03018236	100	Cocaine addiction, alcohol addiction	Alcohol NAC, alcohol placebo, cocaine NAC, cocaine placebo	Evaluate the use of NAC in the treatment of alcohol and cocaine use disorders	Unknown	NA
Antioxidant Replacement Therapy in Patients with Alcohol Abuse	NCT00936000	38	Alcohol abuse	Protandim	Determine the safety and efficacy of in vivo antioxidant replacement therapy on alveolar–capillary barrier function in individuals with a history of chronic alcohol abuse	Completed	NA
A Study of Pleiotropic Pioglitazone Effects on the Alcoholic Lung (APPEAL Study)	NCT03060772	50	Alcoholism	Pioglitazone	Measure the effect of pioglitazone	Terminated due to the fact of COVID-19	NA
NAC in AD	NCT00568087	46	Alcoholism	NAC, placebo	Find out if NAC reduces alcohol drinking and craving	Completed	Reduction in heavy drinking days for both groups
NAC plus Naltrexone for the Treatment of AD	NCT01214083	111	Alcoholism	NAC plus high-dose naltrexone, high-dose naltrexone alone, low-dose naltrexone alone	Determine which of these combinations works better in reducing alcohol drinking	Completed	Reduction in heavy drinking days for all groups

N-acetylcysteine (NAC); alcohol use disorder (AUD); alcohol dependence (AD); topiramate (TPM); post-traumatic stress disorder (PTSD); traumatic brain injury (TBI); bipolar disorder (BD); substance use disorder (SUD); not available (NA).
